# The Impact of the COVID-19 Pandemic on New Lung Cancer Diagnosis in Mureș County, Romania: A 5-Year Retrospective, Comprehensive Study

**DOI:** 10.3390/medicina61091548

**Published:** 2025-08-29

**Authors:** Georgian-Nicolae Radu, Laura Chinezu, Ramona Teodora Cătană, Petre Carabașa, Adela Nechifor-Boilă

**Affiliations:** 1 Pathology Department, Mureș County Clinical Hospital, 540011 Târgu Mureș, Romania; george.radu098@gmail.com; 2 Department of Histology, George Emil Palade University of Medicine, Pharmacy, Science, and Technology of Târgu Mures, 540142 Târgu Mureș, Romania; ramona.catana@umfst.ro (R.T.C.); adela.nechifor-boila@umfst.ro (A.N.-B.); 3 Pathology Department, Institute of Forensic Medicine, 540141 Târgu Mureș, Romania; 4 Pathology Department, Bistrița County Emergency Clinical Hospital, 420094 Bistrița, Romania; carabasa.petre@yahoo.com

**Keywords:** lung cancer, COVID-19, pandemic, Romania, histological subtypes

## Abstract

*Background and Objectives*: Lung cancer (LC) remains a significant global health issue with poor prognosis. The COVID-19 pandemic has caused delays in cancer patient management worldwide. However, its impact on the incidence of LCs in Romania has not yet been discussed. We aimed to evaluate the impact of lockdown restrictions during the COVID-19 pandemic on new LC diagnoses in a Romanian cohort and the potential associations between demographic characteristics and histological features. *Materials and Methods*: This retrospective study analyzed 750 patients with lung tumors diagnosed in the Pathology Department, Mureș County Clinical Hospital, Romania, between 2018 and 2022. The target population was divided in two cohorts: pre-COVID-19 (1 January 2018–15 March 2020) and COVID-19 (16 March 2020–31 December 2022). *Results*: The temporal trend of LC diagnosis followed a descending pattern over the study period, with a significant 72% reduction (*p* < 0.001) in the first year of the COVID-19 pandemic (2020 vs. 2019). In terms of histology, several subtypes displayed a notable reduction in the COVID-19 cohort compared to the pre-pandemic period: squamous carcinoma (SQC) (*p* < 0.001), adenocarcinoma (ADK) (*p* < 0.001), and lung metastases (*p* = 0.0008). On the other hand, cases of non-small-cell lung carcinomas not otherwise specified (NSCLCs NOS) experienced a significant increase in the pandemic years (*p* = 0.0406). SQC was the most frequent subtype of LC and was significantly more frequent in men (*p* < 0.001, RR = 1.3004, 95% CI [1.1786–1.4347]). Furthermore, a notable shift in the male-to-female ratio was observed between the two cohorts, caused by a larger decrease in the incidence of LC among men compared to females in the COVID-19 period (*p* = 0.0002; pre-COVID-19 M/F = 4/1 versus COVID-19 M/F = 2/1). *Conclusions*: COVID-19-related restrictions led to a significant drop in new LC diagnoses during the first year of the pandemic, which was followed by a slight upward trend in the subsequent years. Additionally, the sharp decline in the number of cases among men narrowed the gender gap in LC patients.

## 1. Introduction

According to GLOBOCAN, approximately 100,471 new cases of malignant tumors were reported in Romania in 2022, with the most common sites being the colorectum, breast, and lungs [1]. Worldwide, lung cancer (LC) continues to be the primary cause of cancer-related death for both men and women [2], whereas in our country, LC is the leading cause of cancer-related mortality for males, but it ranks third for females.

In terms of histology, lung malignancies are divided based on the cells of origin into two major categories: non-small-cell lung cancer (NSCLC) and small-cell lung cancer (SCLC). NSCLC is an umbrella term that comprises a wide range of histological subtypes including lung adenocarcinoma (ADK), squamous-cell carcinoma (SQC), and large-cell carcinoma, each one with unique microscopic and molecular characteristics. In recent years, the incidence of ADK exceeded that of SQC, becoming the most frequent histologic subtype in industrialized countries [3]. However, in Romania, this change has not yet been observed. SCLC is a more aggressive subtype that primarily affects smokers, and it accounts for up to 15% of all LC cases [4].

Despite recent technological advances in diagnostic procedures and treatments, most patients have a poor prognosis, especially because up to 75% of LC patients present with advanced stages at the time of diagnosis [5]. Histopathological examination of small tissue biopsies continues to be the gold standard in confirming pulmonary malignancies, relaying on hematoxylin–eosin staining, complemented by immunohistochemistry and, increasingly, molecular profiling.

On 11 March 2020, the World Health Organization (WHO) declared coronavirus disease 2019 (COVID-19) a pandemic [6]. This global event dramatically changed the lives of many, and had a significant impact on how cancer patients were treated. The quarantine restrictions imposed during the COVID-19 pandemic limited patients’ access to healthcare facilities, leading to delays in cancer screening, diagnosis, and treatment.

In Romania, the first COVID-19 case was confirmed in the southwestern region of the country on 26 February 2020 [6]. Considering the constant rise in COVID-19 infections, on 16 March, a state of emergency was declared by the Romanian government, leading to restrictions being imposed on the population, including freedom of movement [6]. The pandemic placed considerable pressure on the regional healthcare systems nationwide, particularly in emergency and intensive care units, while pathology departments implemented standardized procedures to mitigate the risks linked to handling infected tissue samples.

Although the impact of the COVID-19 pandemic on the diagnosis of various solid tumors has been widely investigated, there are limited data in the literature specifically addressing the effects that pandemic-related delays in patients care had on new LC diagnosis.

The aim of our study was to assess the impact of COVID-19 lockdown restrictions on the number of newly diagnosed LC cases at a university hospital in Romania. Additionally, the study offers a comprehensive overview of the epidemiological trends and demographic features of patients diagnosed with LC based on small biopsy specimens within our institution.

## 2. Materials and Methods

### 2.1. Study Design and Data Collection

We performed a 5-year retrospective study including all lung biopsy specimens analyzed in the Pathology Department, Mureș County Clinical Hospital, Târgu Mureș, Romania, between 1 January 2018 and 31 December 2022. The target population was divided into two cohorts based on the day when the Romanian government declared COVID-19 an emergency and implemented lockdown restrictions [6]:

- Pre-COVID 19 cohort (1 January 2018–15 March 2020);
- COVID-19 cohort (16 March 2020–31 December 2022).

Inclusion criteria were (1) a histopathological diagnosis consistent with a neoplastic lesion of the lung, herein including benign, premalignant, and malignant lesions, with malignant lesions accounting for the great majority of cases (LC), and (2) sufficient diagnostic material available on the biopsy specimen to enable a conclusive diagnosis. Lung biopsy cases that were inadequate (insufficient tissue sample or extensive necrosis) or corresponding to inflammatory lung lesions or reactive conditions were excluded from the analysis.

All small lung biopsy specimens that fulfilled the adequacy criteria were included in the analysis. Patients were identified using their individual laboratory registration number. Demographic (age, gender) and pathological data were retrieved from institutional databases and pathological reports. The criteria of the STROBE checklist for observational studies were used in drafting the manuscript.

The study was approved by the Ethics Committee of the Mureș County Clinical Hospital, Târgu-Mureș (letter of approval no. 13/14.01.2023), and by the Ethics Committee of George Emil Palade University of Medicine, Pharmacy, Science, and Technology (letter of approval no. 2086/15.02.2023). Informed consent was obtained from all of the patients included in this study.

### 2.2. Pathological Data

The histopathological diagnosis of various lung neoplasms was established based on their characteristic morphological features and immunohistochemical profiles, in accordance with the 2021 WHO Classification of Thoracic Tumors diagnostic criteria [7]. Cases were divided into 10 distinct groups: (1) SQCs; (2) ADKs; (3) non-small-cell lung carcinomas not otherwise specified (NSCLCs NOS); (4) adenosquamous carcinomas; (5) SCLCs; (6) carcinoids; (7) metastasis; (8) others: malignant; (9) premalignant lesions (squamous dysplasia); and (10) benign tumors.

LC histology was defined in accordance with the 2021 World Health Organization (WHO) Classification of Thoracic Tumors [7], as follows: (1) *SCC* is a tumor consisting of sheets of tumor cells with abundant eosinophilic cytoplasm, enlarged hyperchromatic nuclei, one or two nucleoli, and a clearly visible intercellular border. Hallmarks of squamous differentiation included keratin pearl formation and intercellular bridges. Immunohistochemically, the tumor cells exhibited nuclear positivity for p40. (2) A diagnosis of *ADK* was set based of characteristic morphological features (glandular, acinar, papillary, micropapillary, solid, fetal, or enteric pattern), supported by positive TTF1 nuclear staining whenever the morphology was not conclusive. (3) *SCLC* was defined by sheets or nests of small-to-medium-sized cells with a neuroendocrine phenotype, scant basophilic cytoplasm, and irregular, hyperchromatic nuclei, as well as evidence of crush artifacts present in most cases; the tumor cells revealed a high Ki-67 index. (4) Cases of NSCLCs NOS corresponded to poorly differentiated carcinomas without morphological and immunohistochemical evidence of neither squamous nor glandular differentiation.

Our study included only histopathological data since information regarding molecular pathology (genetic mutations) were not available.

### 2.3. Statistical Analysis

All statistical analyses were conducted using Epi Info version 7.2.0.1, GraphPad Prism version 10.5.0, and Microsoft Excel version 16.93. Descriptive statistics were used to characterize the population groups. All data collected in the study were labeled as categorical variables (sex, histological subtype, grade of dysplasia) or quantitative variables (age, year). Nominal variables were summarized as counts and frequencies. The distributed continuous variables were expressed as mean +/− standard deviation (SD).

The normal distribution of continuous variables was assessed using the Kolmogorov–Smirnov test. The frequencies of the nominal and categorical variables were compared using the Chi-squared test, and subsequently, risk ratios (RRs) and corresponding 95% confidence intervals (CIs) were calculated. We analyzed the data in a comparative way between two periods: pre-COVID-19 (1 January 2018–15 March 2020) and during the COVID-19 pandemic (16 March 2020–31 December 2022). To compare the change in distribution between the two cohorts, the Z test was applied.

To assess associations, simple logistic regression models were fitted. The likelihood ratio test (LRT) was used to evaluate statistical significance. Odds ratios (ORs) with corresponding 95% confidence intervals were reported. Model fit was assessed using Tjur’s R squared.

The analysis of the temporal trend variations of lung tumors (LTs) was performed using JOINPOINT version 5.3.0 software. We used annually collected LT incidence data for each gender and age category, which were age-adjusted based on population rates provided by the Romanian National Institute of Statistics. Subsequently, the software calculated the annual percentage change (APC) with 95% confidence intervals in order to identify significant trend variations.

We categorized changes in quantitative variables as follows: mild (+/− 0–33.33%), moderate (+/− 33.34–66.66%), and marked (+/− 66.67–100%).

The level of statistical significance was set at *p* < 0.05. Bonferroni correction was applied to adjust *p*-values in order to reduce the probability of type I errors.

## 3. Results

### 3.1. Patients’ Characteristics

**Figure 1:**
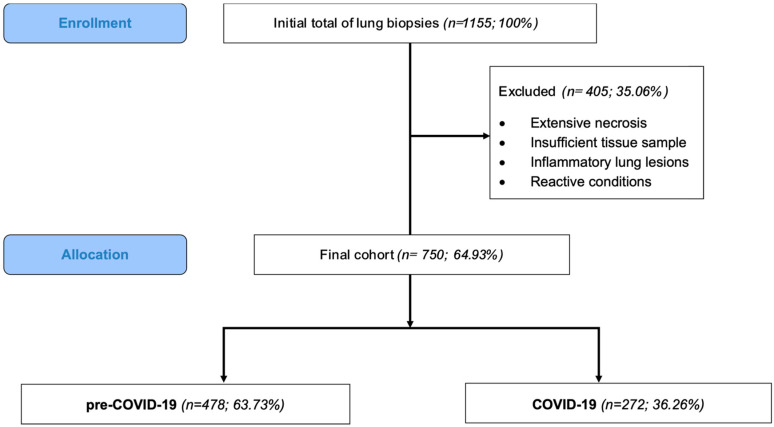
Selection of cases and study cohort stratification. Out of 1155 registered lung biopsy specimens, 405 biopsies did not meet the inclusion criteria and were excluded (due to extensive necrosis, insufficient tissue sample, inflammatory lung lesions, or reactive conditions). The final cohort consisted of 750 cases, which were divided into two groups: pre-COVID-19 (*n* = 478; 63.73%) and COVID-19 (*n* = 272; 36.26%). A significant 43.1% decrease in the number of cases was observed between the two groups (*p* < 0.001).

Out of the 1155 lung biopsy specimens registered in our department throughout the 5-year time frame (2018–2022), 750 (64.93%) cases fulfilled the inclusion and exclusion criteria and were further considered in the analysis; 63.73% of cases (*n* = 478) corresponded to cases registered in the pre-COVID-19 period (1 January 2018–15 March 2020), while the remaining 36.26% (*n* = 272) cases were recorded during the COVID-19 period (16 March 2020–31 December 2022) (Figure 1).

The distribution of cases by gender, age, and histopathological diagnosis in the pre-COVID-19 versus COVID-19 period is illustrated in Table 1.

Among the patients registered in the pre-COVID-19 group, the majority (*n* = 383, 80.13%) were males, and only 19.87% (*n* = 95) were females (Table 1); the mean age at diagnosis was 65.51 ± 9.42 years old (range 30–86). Regarding age distribution, the majority of lung neoplastic lesions occurred in patients older than 60 years old (*n* = 351, 73.43%); only 26.56% (*n* = 127) of patients were younger than 60 years old in this group.

Gender distribution revealed a similar pattern over the pandemic period (in the COVID-19 cohort), with men accounting for most of the cases (*n* = 185, 68.01%) while the number of cases in women was significantly fewer (*n* = 87, 31.99%). The mean age at diagnosis in the COVID-19 cohort was 65.59 ± 9.41 years old (range 35–87). With regard to age distribution, similar results to the pre-COVID-19 cohort were observed, with the majority of the patients being older than 60 years old (*n* = 199, 73.16%) in this second group as well.

With regard to the male-to-female (M/F) ratio, the number of females remained relatively constant over the two study periods (*n* = 95, 19.87% in the pre-COVID-19 group and *n* = 87, 31.99% in the COVID-19 group). A significant reduction in the number of males (52%, *p* < 0.001) was observed in the second study period, which has led to a significant shift in M/F ratio in the pandemic years (M/F: 4/1 in the pre-COVID-19 period versus M/F: 2/1 in the COVID-19 period, *p* = 0.0002) (Table 1).

### 3.2. Pathological Data

Regarding histopathological diagnosis, SQCs accounted for approximately one third of all the cases (*n* = 257; 34.27%), followed by ADK (*n* = 219; 29.20%) and SCLC (*n* = 106; 14.13%) (Table 1). Other types of lung neoplasms (NSCLC NOS, adenosquamous carcinoma, carcinoids, metastases, benign tumors, and squamous dysplasia) were less frequent.

The changes in the frequency of different histopathological diagnoses between the two study groups are illustrated in Table 1. When the two study periods were analyzed in comparison, the following findings were observed: a mild reduction in the benign category (-33.33%); a moderate reduction in SQCs (−47.9%, *p* < 0.001), ADKs (−44.6%, *p* < 0.001), SCLCs (−29%), and carcinoid tumors (−60%); and a marked reduction in squamous dysplasia (−79.4%, *p* = 0.0004) and lung metastases (−82.7%, *p* = 0.0008) in the COVID-19 period versus pre-COVID-19 period.

With regard to secondary lung tumors, out of the 34 cases, 29 (85.3%) were registered before the COVID-19 pandemic and only 5 (14.7%) cases were recorded during the pandemic years. In females, the primary sites of origin were as follows: breast (*n* = 9; 52.95%), gastrointestinal tract (*n* = 4; 23.52%), gynecological cancer sites (*n* = 3; 17.64%), and urinary tract (*n* = 1; 5.88%). Among male patients, the most common site of origin for lung metastasis was the gastrointestinal tract (*n* = 10; 58.82%), followed by the skin (*n* = 2; 11.76%) and urinary tract (*n* = 1; 5.88%), while in 4 cases (23.52%), the primary site of origin could not be determined.

### 3.3. Temporal Trend

**Figure 2:**
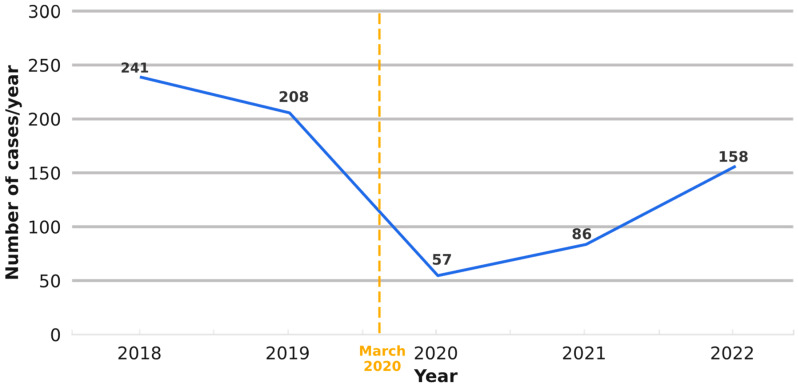
Number of lung neoplasms diagnosed in our department/year and their time trend evolution over the entire study period (between 2018 and 2022). The most substantial drop in the number of lung neoplasms occurred in 2020 (*n* = 57), corresponding to a 72% reduction compared to 2019.

Our data have revealed a descending trend in the annual diagnostic volume of lung neoplasms diagnosed in our department over the entire study period (between 2018 and 2022) (Figure 2). The most substantial drop in the number occurred in 2020 (*n* = 57), corresponding to a 72% reduction compared to 2019 (*p* < 0.001). The annual number of cases increased in 2021 (+29 cases [51%]) and 2022 (+72 cases [83%]), respectively, but still remained lower than in 2018 (Figure 2).

**Figure 3:**
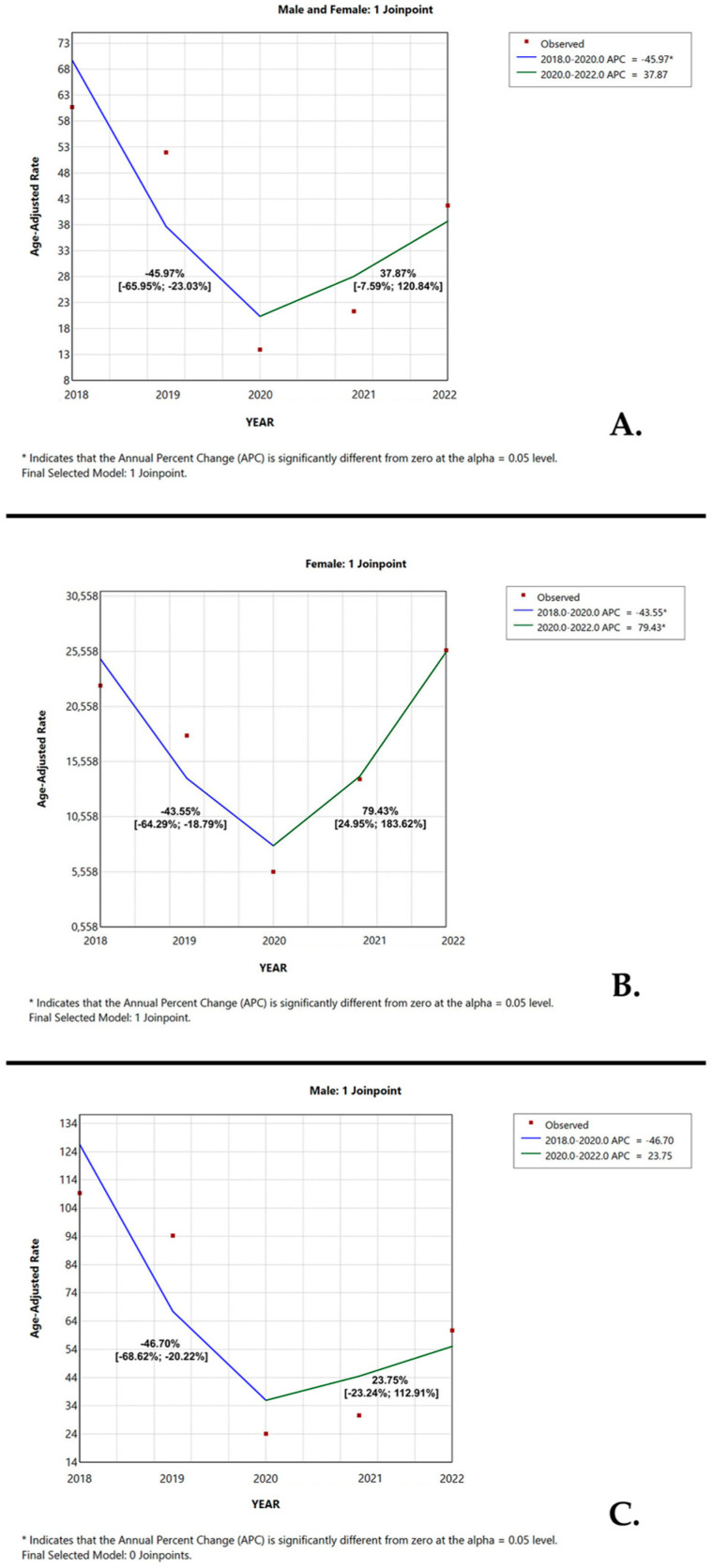
Temporal trend of lung neoplasms’ incidence between January 2018 and December 2022, stratified by sex. (**A**) For males and females, there is a downward trend with a significant change in the annual percentage change (APC) of −45.97% (95% CI [−65.95%; −23.03%], *p* < 0.001) in 2020, which was followed by an upward trend. (**B**) For females, there is a significant descending trend between 2018 and 2020 with an APC of −43.55% (95% CI [−64.29%; −18.79], *p* = 0.0012) followed by a significant increase between 2020 and 2022 with an APC of +79.43% (95% CI [24.95%; 183.62%], *p* < 0.001). (**C**) For males, there is a descending trend between 2018 and 2020 with an APC of −46.70% (95% CI [−68.62%; −20.22%]) which was followed by a slight increase during 2020–2022 with an APC of +23.75 (95% CI [−23.24%; 112.91%]).

Figure 3 illustrates the APC in age-adjusted diagnosis volume of lung neoplasms between 2018 and 2020, further dichotomized by sex. Based on the joinpoint analysis, the lung neoplasms’ diagnostic rates over the entire study period showed a significant downward trend with an APC of −45.97% (95% CI [−65.95%; −23.03%], *p* < 0.001) from 2018 to 2020. This was followed by an increase in the APC of +37.87% (95% CI [−7.59%; 120.84%], *p* = 0.1135) from 2020 to 2022 (Figure 3A).

When dichotomized by gender, lung neoplasms’ frequency among females displayed a significant decline in the first study period (2018–2020) with a change in APC of −43.55% (95% CI [−64.29%; −18.79], *p* = 0.0012). This was subsequently followed by a rise in the APC to +79.43% (95% CI [24.95%; 183.62%], *p* < 0.001) in the second half of the study period (Figure 3B). Over the same time frame, the case volume of lung neoplasms in males exhibited the greatest variation in 2020, with a descending trend between 2018 and 2020 (APC of −46.70%, 95% CI [−68.62%; −20.22%]). This was followed by an upward trend in the subsequent years (APC of +23.75%, 95% CI [−23.24%; 112.91%]) (Figure 3C).

### 3.4. Associations Between Demographic and Morphologic Characteristics of the Study Cases

Demographic and morphologic characteristics of the cases were compared to identify potential links. Specifically, we compared gender and age (with a focus on patients >60 years old) to histological subtypes of LC (SQCs, ADKs, SCLCs, and metastases, respectively). Male gender was found to be a risk factor for SQCs (*p* < 0.001, RR = 1.3004, 95% CI [1.1786–1.4347]), in contrast to lung metastases, where male gender was associated with a protective role (<0.001, RR = 0.9346, 95% CI [0.8900–0.9813]). Even though men outnumbered females in both ADK and SCLC cases, we did not find any significant association between male gender and these morphologic subtypes of LC (ADK: *p* = 0.07, RR = 0.9014, 95% CI [0.8017–1.0135]; SCLC: *p* = 0.097, RR = 0.9394, 95% CI [0.8713–1.0129]).

Patients older than 60 years old were the most numerous in our study population. No significant associations were observed between age (>60 years old) and histological subtypes of LC: SQC (*p* = 0.995, RR = 1.0055, 95% CI [0.8950–1.1297]); ADK (*p* = 0.283, RR = 1.0627, 95% CI [0.9625–1.1732]); SCLC (*p* = 1, RR= 1.0021, 95% CI [0.9386–1.0699]); and metastases (*p* = 0.863, RR = 0.9933, 95% CI [0.9578–1.0302]).

In order to assess the association between demographic characteristics (age and sex) and histologic subtypes, simple logistic regression models were applied. Male sex was associated with significantly higher odds of developing SQC (OR = 2.477, 95% CI: 1.679–3.733, *p* < 0.001) and significantly lower odds of developing lung metastases (OR = 0.2995, 95% CI: 0.1487–0.6030, *p* < 0.001) compared to females. However, gender explained only 2.76% of the variance in SQC and 1.71% in metastases, as indicated by Tjur’s R squared. The other variables were not statistically significant and are presented in Table 2.

## 4. Discussion

Since March 2020, the COVID-19 pandemic has dramatically transformed cancer care and diagnosis and left a lasting impact on the global healthcare landscape. Romania, like every other country, had limited access to in-person care during the pandemic years, which affected patients’ ability to obtain proper and rapid cancer diagnosis [8,9].

LC is a major public health concern that seriously threatens the health of the global population, particularly because it is oligosymptomatic until advanced stages [10,11], posing challenges in early detection strategies. LC incidence is projected to increase by approximately 50% in males and nearly double in females by 2035 [12]. During pandemic years, oncologic patients faced difficulties in screening and diagnostic procedures, resulting in reduced incidence of numerous malignancies including LC, compared to pre-pandemic years [13]. Moreover, it is believed that LC itself represents an independent mortality risk factor for COVID-19 patients [14].

This current study focuses on the impact of the COVID-19 pandemic period on the time trend prevalence of pulmonary neoplasms in small biopsy specimens in a university hospital in Romania (Mures County Clinical Hospital), with special emphasis on the diagnosis of LC. Furthermore, we investigated the potential links between gender, age, and histologic subtypes, particularly NSCLC (SQC and ADK), SCLC, and lung metastases. To the best of our knowledge, this is among the first studies to investigate the clinico-pathological characteristics of LC in a Romanian cohort, focusing on the COVID-19 pandemic period.

The impact of the COVID-19 pandemic on new cancer diagnoses has been widespread and well documented across the globe. During the study period, we observed a downward trend in LC diagnoses, with a marked impact during the pandemic years. The greatest fluctuation in this trend occurred during the COVID-19 period (from 16 March 2020 to December 2022). Our findings indicate that disruptions in cancer care for Romanian patients due to pandemic-related restrictions led to a significant 72% decline in the diagnosis of new lung neoplasms during the first year of the COVID-19 pandemic. However, following this initial reduction, new diagnoses displayed an increase by 51% in the second pandemic year and by 83% in the third pandemic year, respectively. Similarly, research conducted in Italy, Canada, and the United States reported relevant decreases in LC incidence in the first year of the COVID-19 pandemic (−48% in Italy; −34.7% in Canada; −75% in the United States) [15,16,17]. However, a slight-to-moderate reduction (between 7.5% and 21.5%) in newly diagnosed LC cases was observed in several other studies [18,19,20,21].

Romania’s healthcare infrastructure (limited preventive medicine and restricted accessibility in underdeveloped regions), combined with insufficient logistic and strategic interventions (fewer screening programs), created major vulnerabilities. These factors, together with widespread public mistrust during the lockdown period, likely contributed to the pronounced decline in LC diagnoses observed during the pandemic. In contrast, other European countries with well-established and efficient patient–clinician communication strategies (like telemedicine and multiprofessional community health centers) [22] reported only minor reductions. Conversely, the magnitude of these reductions may have varied according to differences in regional pandemic timelines. Notably, these systemic shortcomings, together with the complete suspension of non-urgent medical services, underscore the substantial reduction in the diagnosis of different solid tumors across multiple institutions nationwide (cervical cancer (−45%), melanoma (−19.3%), hepatocellular carcinoma (−31.91%)) [23,24,25].

The decline in cancer diagnoses can be attributed to multiple factors, involving both patients and the healthcare system. Fear of contracting COVID-19 has limited patients’ access to medical services and decreased their willingness to report symptoms [26,27]. Limited access to healthcare has led to major disruptions in healthcare delivery, with potential long-term consequences for patients who missed crucial cancer screening, diagnosis, and treatment. These delays in diagnosis may have allowed LC to progress to more advanced stages, increasing the risk of higher mortality rates and long-term health complications [28]. Subsequently, Mangone et al. reported an increase in advanced and metastatic LC following lockdown measures in Italy [29].

Regarding histologic subtypes, SQC remains the most frequent subtype in our region, in line with previous studies across the country [30,31]. On the contrary, at a global level, the incidence rates of ADK exceeded those seen in SQC in recent years [3,5]. This trend is more likely associated with changes in cigarette design and composition, as well as genetic predisposition and environmental exposure in female never-smokers [32]. According to the Global Adult Tobacco Survey (GATS) 2018, 30.7% of the Romanian population smokes, with an average age of daily smoking initiation of 17.9 years [33]. Since SQCs are strongly associated with tobacco use, this high prevalence may explain why Romania deviates from the global trend of ADK predominance. In addition, SQCs are typically located centrally, near the main bronchi, and are more easily accessible by bronchoscopy, whereas ADKs are usually peripheral and more often sampled via transthoracic biopsy. As our study included only endobronchial tissue samples, this sampling bias may have contributed to the higher proportion of SQCs observed.

We also explored how the incidence trends of different histologic subtypes of lung neoplasms have evolved during the COVID-19 pandemic. In line with the existing literature, we observed a reduction in new cases of SCLC, SQC, and ADK [9,29]. However, to our knowledge, previous studies have not expanded their analysis to include other histologic subtypes of pulmonary lesions. Our research reveals a drop in diagnosis volume of carcinoid tumors, benign lesions, and adenosquamous carcinomas, with a significant decline in lung metastases (*p* = 0.0008) and squamous dysplasia (*p* = 0.0004). Interestingly, the sole exception to this downward trend was NSCLCs NOS, which exhibited a notable increase in number of cases.

Some possible explanations for the observed increase in NSCLC NOS cases during the pandemic period include the small size of biopsy samples (less than 1 mm), which often restricts immunohistochemistry panels to four or five markers (TTF1, CK7, p40, CK AE1/AE3, and CD56). The absence of molecular profiling may also have contributed to the rise in NSCLC NOS diagnoses. In addition, severe acute respiratory syndrome coronavirus 2 (SARS-CoV-2)-related lung disease and sampling difficulties caused by infection prevention measures may have resulted in inadequate biopsy material and increased diagnostic uncertainty among pathologists. On the other hand, the sharp decline in lung metastases may be attributed to reduced screening programs and delays in diagnostic workups during the COVID-19 pandemic, potentially resulting in more advanced stages of different types of solid tumors in the future. However, this hypothesis requires further investigation in future studies.

In 2020, lung neoplasm cases declined in both sexes but this was more pronounced among males, resulting in a relative increase in female cases. This shift revealed a significant trend (*p* = 0.0002) in the demographic distribution of LC patients, which began prior to the COVID-19 pandemic but was notably influenced by it. Nevertheless, these findings require confirmation through multicenter studies across Romania. This gender gap shift may reflect the smoking patterns reported in GATS 2018. At that time, 40.4% of Romanian men and 21.7% of Romanian women were current tobacco users, with a slightly greater increase in female smokers (+5%) compared with males (+3%) since GATS 2011 [33,34]. At a global level, Mangone et al. reported similar results and stated that the fluctuation in trend is most probably linked to increasing tobacco consumption among the female population [29]. Prospective models indicate that the gender gap in LC incidence is gradually narrowing, with some projections suggesting that by 2035, the incidence of LC in women will reach the same level as that among men [12]. There is conflicting evidence on whether women are more prone to developing LC. Some genetic factors such as CYP1A1 over-expression, p53 mutations, and early gastrin-releasing peptide receptor (GRPR) activation and over-expression have been postulated to increase the risk of LC among females [35].

Several studies have reported associations between histological subtypes of LC and patients’ sex. These studies found that the SQC histology is more likely to appear in males while ADK tends to occur more frequently in females [35,36,37]. Similarly, our study showed a strong association between the male sex and SQC (*p* < 0.001). Since SQC is highly linked to smoking habits, this result could be explained by the high prevalence of tobacco consumption among the Romanian population, specifically Romanian men. According to GATS 2018, around 5.5 million Romanian adults aged 15 and above admitted to smoking (among which 40% were male) [33,38].

Lung metastases are widely recognized for their poor prognosis in cancer patients. Chen et al. found that patients with synchronous lung metastasis were typically older males who had more advanced T- and N-stages at the time of diagnosis [39]. Similarly to carcinogenesis, the molecular biology of metastasis involves a stepwise process that enables cancer cells to acquire specific characteristics required for dissemination [40]. Interestingly, in contrast to other research work, we found that men have a slightly lower chance of developing lung metastases compared to women (*p* < 0.001).

LC is typically diagnosed in adulthood, with our data showing that most cases occur in individuals over 50 years of age (94.1%). From these data, the highest number of patients fell within the 61–70 age range (43% of men and 40.1% of women). The average age at the time of diagnosis remained consistent before and during the pandemic years (pre-COVID-19: 65.51 ± 9.42 years; COVID-19: 65.59 ± 9.41 years). In a similar manner, at a global level, LC is being diagnosed in individuals over 60 years of age, with an average age at diagnosis of 70 years [38]. Likewise, studies conducted in Romania also reported that the majority of LC cases occur in the 60–70 age group, with a male sex predominance [30,31].

Carcinogenesis represents a multistage process that requires a sequential accumulation of DNA alterations within cells as a result of exposure to intrinsic and extrinsic risk factors [41]. However, genomic instability is considered a hallmark of both cancer and senescence. Thus, the susceptibility for LC in the older population is attributed to genetic mutations due to continuous exposure to carcinogens (tobacco smoke and air pollution) combined with a decline in immune function and increased age-related cellular changes [42]. However, despite this, our findings did not reveal any clear association between age and specific LC subtypes. On the other hand, LC rarely occurs in younger adults, with only 1.47% of lung tumors found in patients under 40 years old in our study.

Several limitations of our study should be acknowledged. This was a single-center study with a moderate sample size, relying solely on small tissue samples (excluding cytology, resection specimens, and inadequate biopsy samples (with extensive necrosis, insufficient tissue sample, inflammatory lung lesions, or reactive conditions)). Moreover, potential bias might have arisen from a higher-than-usual rate of inadequate biopsies, largely attributable to the learning curve of newly trained pulmonologists and the predominance of necrotic, nonviable tissue that precluded definitive diagnosis. Additionally, we did not incorporate molecular profiling (e.g., BRAF, EGFR, KRAS, MET, or NTRK), survival status, or tumor stage data into our analysis, so we could not directly evaluate the prognostic impact of the COVID-19 pandemic on different histologic and molecular subtypes of LC in our region. However, we have highlighted this as a priority for future research.

## 5. Conclusions

Our data reveal that SQC remains the most frequent histologic subtype of LC diagnosed from biopsy specimens in our region. In our study group, most patients diagnosed with LC were men older than 60 years old. Nevertheless, recent years have seen a shift towards a relative increase in the number of cases among women, as a result of a sharp decline in male cases.

The COVID-19 pandemic has had a dramatic impact on the Romanian healthcare system, causing delays in the diagnosis and treatment of oncologic patients, including those with LC. Following the implementation of lockdown restrictions in March 2020, new LC diagnoses dropped by 72% in the first year of the pandemic, then nearly doubled in the subsequent year. The long-term impact of this global event on LC patient outcomes remains to be seen and requires further confirmation through larger prospective studies.

## Figures and Tables

**Table 1:** The demographic characteristics and histologic diagnosis of the cases in comparison between the two cohorts (pre-COVID-19 and COVID-19).

Characteristics	pre-COVID-19 (*n* = 478)	COVID-19 (*n* = 272)	*p* *
Sex *n* (%)			
Females	95 (19.87%)	87 (31.99%)	-
Males	383 (80.13%)	185 (68.01%)	**-**
M/F ratio	4/1	2/1	0.0002 **
Age			
Mean	65.51 ± 9.42	65.59 ± 9.41	
≤40	8 (1.67%)	3 (1.10%)	0.17
41–50	20 (4.18%)	13 (4.78%)	0.23
51–60	99 (20.71%)	57 (20.96%)	**0.0012**
61–70	201 (42.05%)	116 (42.65%)	***p *** **< 0.001**
>70	150 (31.38%)	83 (30.51%)	***p *** **< 0.001**
Histology *n* (%)			
Squamous carcinoma	169 (35.36%)	88 (32.35%)	***p *** **< 0.001**
Adenocarcinoma	141 (29.50%)	78 (28.68%)	***p *** **< 0.001**
Non-small-cell carcinoma NOS	14 (2.93%)	28 (10.29%)	0.0406
Adenosquamous carcinoma	2 (0.42%)	0 (0%)	-
Small-cell carcinoma	62 (12.97%)	44 (16.18%)	0.0845
Carcinoids	5 (1.05%)	2 (0.74%)	0.2965
Metastases	29 (6.07%)	5 (1.84%)	**0.0008**
Others: benign	6 (1.26%)	4 (1.47%)	0.5351
Others: malignant	16 (3.35%)	16 (5.88%)	1
Squamous dysplasia	34 (7.11%)	7 (2.57%)	**0.0004**

* The *p*-value was obtained by comparing the increase/decrease in number of cases between the two groups; a Z test was performed; statistically significant differences are shown in bold. Bonferroni correction was applied, yielding a revised significance threshold of α = 0.003. ** The *p*-value for comparing the M/F ratio between the two cohorts was obtained performing a Chi-squared test.

**Table 2:** Logistic regression results for the association between demographic characteristics (age and sex) and histology.

	OR	95% CI	Tjur’s R Squared	*p* *
SQC				
Gender	2.477	1.679–3.733	0.02763	**<0.001**
Age > 60 years	1.016	0.7242–1.435	0.00001148	0.926
ADK				
Gender	0.7152	0.5018–1.025	0.004545	0.0678
Age > 60 years	1.241	0.8662–1.798	0.001801	0.2416
SCLC				
Gender	0.6659	0,4276–1,055	0.004221	0.0822
Age > 60 years	1.015	0.6441–1.640	0.000005327	0.9496
Metastases				
Gender	0.2995	0.1487–0.6030	0.01711	**<0.001**
Age > 60 years	0.8669	0.4184–1.930	0.000183	0.7138

* The *p*-value was obtained performing the likelihood ratio test (LRT) for logistic regression models. Bonferroni correction was applied, yielding a revised significance threshold of α = 0.006. Statistically significant differences are shown in bold.

## Data Availability

The data presented in this study are available on request from the corresponding author. The data are not publicly available due to ethical restrictions (personal data protection of the patients included in the study).

## Abbreviations

ADK	Lung adenocarcinoma
APC	Annual percentage change
BRAF	V-Raf Murine Sarcoma Viral Oncogene Homolog B
CD 56	Cluster of Differentiation 56
COVID-19	Coronavirus disease 2019
CYP1A1	Cytochrome P450 Family 1 Subfamily A Member 1
CK AE1/AE3	Cytokeratin AE1/AE3
CK 7	Cytokeratin 7
DNA	Deoxyribonucleic acid
EGFR	Epidermal growth factor receptor
GATS	Global Adult Tobacco Survey
GRPR	Gastrin-releasing peptide receptor
KRAS	Kirsten rat sarcoma virus
LC	Lung cancer
LT	Lung tumor
MET	Mesenchymal–epithelial transition factor
M/F	Male-to-female ratio
NSCLC NOS	Non-small-cell lung cancer not otherwise specified
NSCLC	Non-small-cell lung cancer
NTRK	Neurotrophic tyrosine receptor kinase
p40	Nuclear marker with expression in squamous-cell differentiation (p63 isoform)
p53	Tumor suppressor gene at 17p13
RR	Relative risk
SCLC	Small-cell lung cancer
SD	Standard deviation
SQC	Squamous-cell carcinoma
WHO	World Health Organization
TTF1	Thyroid transcription factor 1; nuclear marker
